# Financing the introduction of new vaccines to the national immunisation programme in China: challenges and options for action

**DOI:** 10.1136/bmjgh-2024-017970

**Published:** 2025-03-24

**Authors:** Xinyu Zhang, Shu Chen, Kun Zhu, Shenglan Tang

**Affiliations:** 1 Global Health Research Center, Duke Kunshan University, Kunshan, Jiangsu, China; 2 Duke Global Health Institute, Duke University, Durham, North Carolina, USA; 3 Chinese Academy of Fiscal Sciences, Beijing, China; 4 SingHealth Duke-NUS Global Health Institute, Duke-NUS, Singapore

**Keywords:** Vaccines, Health policy, Immunisation, Public Health

## Abstract

Ensuring adequate and sustainable financing of national immunisation programmes (NIPs) is one of the important elements to achieve the Immunisation Agenda 2030. Many middle-income countries ineligible for support from the Global Alliance for Vaccines and Immunisation have relatively slow progress in introducing new critical vaccines, due largely to financial constraints. China has not introduced any vaccines into the NIP since 2008. Its funding for the NIP, relying solely on the government budget, has been decreasing as the number of targeted children has declined. This paper presents the current situation of NIP and identifies main challenges in vaccine introduction in China: fluctuating and insufficient financing, restrictions on using health insurance funds for immunisation, high prices for non-NIP vaccines and the complicated and non-transparent decision-making mechanism to adjust NIP. There are also opportunities for introducing vaccines, such as local pilots to provide free or subsidised new vaccines and reducing domestic vaccine prices. Feasible options to optimise NIP financing in China include increasing government funding, diversifying financing channels such as using health insurance funds, improving the vaccine procurement mechanism and optimising the new vaccine introduction decision-making process.

**SUMMARY BOX**

- As a typical middle-income country ineligible for external support, China has not introduced any new vaccines into the national immunisation programme (NIP) since 2008 due largely to financial constraints.
- Main challenges for financing the new vaccine introduction of NIP in China include decreasing funding from the government and lower investment compared with other countries, restrictions on using health insurance funds for immunisation, high prices for non-NIP vaccines and the complicated and non-transparent decision-making mechanisms about the adjustments of the NIP.
- Opportunities for introducing new vaccines into the NIP in China are, among others, a political commitment to expanding NIP vaccines, local initiatives developed to provide free or subsidised new vaccines and declined domestic-made vaccine prices due to the competition of Chinese manufacturers and the improved procurement mechanisms.
- Feasible options to optimise NIP financing in China may consist of increasing government funding, diversifying financing channels such as using health insurance funds, improving the vaccine procurement mechanisms and optimising the new vaccine introduction decision-making process.

## Introduction

Immunisation is one of the most cost-effective health interventions and the best uses of public finance to provide health benefits for people.^1^,^3^ Given its life-saving nature and positive externalities, the government is expected to undertake the primary financial responsibility to ensure new vaccine introduction and universal vaccine coverage.^4^,^6^ However, securing adequate and sustainable immunisation financing has been identified as one of the key challenges to achieve the Immunisation Agenda 2030,^7^ especially for middle-income countries (MICs) that are ineligible for external support from international donors such as Gavi, the Global Alliance for Vaccines and Immunisation. Compared with their low-income counterparts, many MICs have been relatively slow to introduce new vaccines into their national immunisation programmes (NIPs) due to the limited funding and high vaccine prices.^8 9^ Consequently, vaccine coverage and health outcomes of infecting vaccine-preventable diseases (VPDs) in these MICs are often not as good as low-income countries that have received significant external support.^10^ While Gavi is aware of the issue and established a new MICs Approach in 2020 to prevent MICs from backsliding after graduation, the countries on the support list are still quite limited.^9 11^

China is a typical example of these Gavi-ineligible MICs. It has faced daunting challenges in financing the NIP expansion. China’s NIP has made great achievements over the past four decades in reducing the disease burden of VPDs.^12 13^ Yet, considerable bottlenecks remain—China has not introduced any new vaccine into NIP since 2008,^14^ although many are being highly recommended for all children in routine immunisation by WHO,^15^ including Haemophilus influenzae type b (Hib) vaccine, human papillomavirus (HPV) vaccine, pneumococcal conjugate vaccine (PCV) and rotavirus vaccine. Notably, China is the only WHO member state that has not introduced the Hib vaccine.^16^ The barrier is not related to no licensed domestic vaccines—all these non-NIP vaccines are available in the private sector with domestic products except for 4-valent and 9-valent HPV vaccines. Instead, one of the key barriers to NIP expansion is the financial constraint of the Chinese government, particularly under the slowdown of economic growth in recent years.^17^

Against this background, this paper aims to present an overview of NIP financing, analyse and discuss main challenges, identify opportunities for financing the new vaccine introduction into NIP in China and provide options for action to the government. The findings of this study could also contribute to NIP financing in other MICs facing similar challenges.

## NIP and its financing in China

Since being launched in 1978, the NIP in China has expanded gradually, and it covers 13 vaccines to prevent 12 VPDs for all children under 6 years old as routine immunisation currently.^12 18^ After 2008, the immunisation schedule has been adjusted several times, but no new VPDs have been brought into the list of NIP. Four out of 10 WHO-recommended routine vaccines for children are not included. Adults in China are not included in the national routine immunisation programme.

Vaccines included in the NIP in China are also referred to as Category One vaccines. The government provides them for free for all children regardless of family socioeconomic status.^19^ The Vaccine Administration Law of China stipulates that it is an obligation for the targeted population to receive vaccination.^20^ Non-NIP vaccines, known as Category Two, are optional and paid for out-of-pocket.^21^

**Figure 1:**
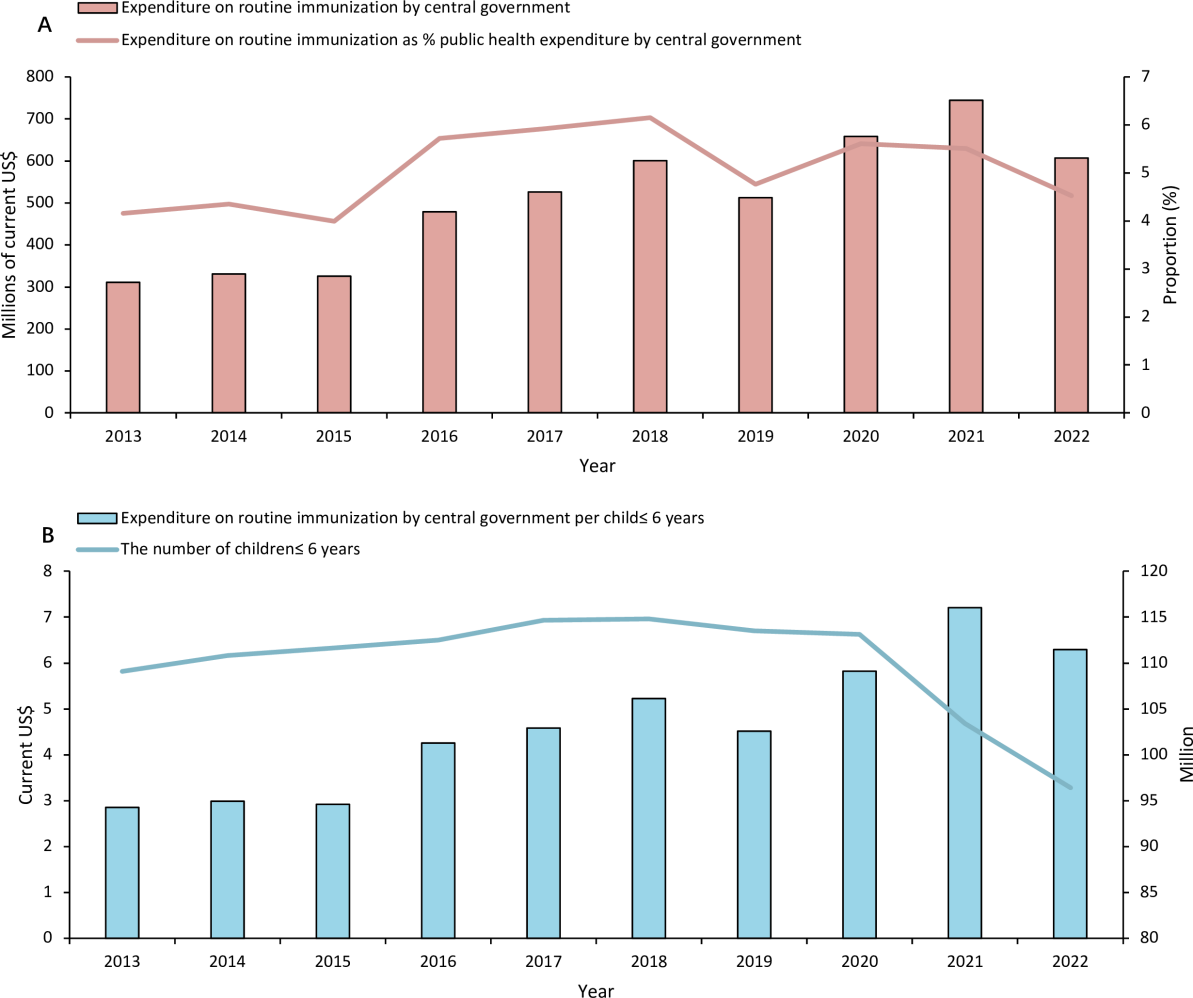
Expenditure on routine immunisation by the central government of China, 2013–2022. In panel A, bars of expenditure on routine immunisation by the central government were plotted on the left vertical axis. The line of the proportion of expenditure on routine immunisation in the central government’s public health expenditure was plotted on the right vertical axis, which was calculated by the expenditure on routine immunisation by the central government divided by the total public health expenditure by the central government. In panel B, bars of expenditure on routine immunisation by the central government per child under 6 years old were plotted on the left vertical axis. The line of the number of children under 6 years old was plotted on the right vertical axis. The number of children under 6 years old was collected from the China Population and Employment Statistical Yearbook (2023) and China Statistical Yearbook (2023).

The expenditure on routine immunisation by the central government of China, the dominant source of NIP financing,^22^ doubled from US$311 million in 2013 to the peak of US$745 million in 2021 but decreased in 2022 to US$607 million (figure 1A). Local governments at different administrative levels co-fund the operation of NIP through earmarked or essential public health service funds.^19^ All vaccines included in the NIP are domestically manufactured, and the prices are determined by joint bidding and procurement at the national level.^12 21^

## Main challenges for financing the new vaccine introduction

### Fluctuant and insufficient financing

Though NIP financing by the central government has been increasing generally in the past decade, the trend was fluctuating, and the spending has decreased since 2022 (figure 1A; see box 1). It was estimated to further decrease to US$494 million in 2023. This is associated with the declining birth population—the number of NIP-covered children decreased from 109.11 million in 2013 to 96.42 million in 2022 (figure 1B). In fact, the birth rate in China has been declining significantly since 2018,^23^ causing a lagging decrease in NIP funding since 2019. A temporary increase in 2020 and 2021 was mainly due to the investment in COVID-19 vaccines. As a result, the investment of the central government on routine immunisation per child under 6 years old increased from US$2.85 in 2013 to the peak of US$7.20 in 2021, then decreased to US$6.30 in 2022 (figure 1B).

Box 1 **Data sources of this study**

We collected quantitative data on the level and historical trend of national immunisation programme (NIP) financing and public health expenditure in China from the Ministry of Finance, China. We also used a comparative approach to analyse the quantitative data from the WHO Immunisation expenditure data after assessing the comparability, data quality and availability. We selected several Gavi, the Vaccine Alliance-ineligible middle-income countries with similar economic development status to China for comparison, that is, Philippines, Mexico, Thailand, South Africa and Brazil, and some high-income countries with well-performing NIPs, that is, the Netherlands, Australia and South Korea for China’s reference. All these countries fully finance the routine immunisation expenditure with government funds like China in recent years. Due to the incomplete historical immunisation financing data, we used each country’s most recent available data.

Qualitative data on the challenges and opportunities of NIP financing were collected from semistructured interviews with experts and key informants from the Ministry of Finance of China, the National Disease Control and Prevention Administration, the Center for Disease Control and Prevention (CDC) of China, the National Healthcare Security Administration and local CDC, finance and health authorities.

The proportion of expenditure on routine immunisation in the central government’s public health expenditure has been continuously decreasing over the past 3 years (figure 1A). This share of funding allocated to immunisation is affected by competing priorities, typically other vertical public health programmes.^5^ In China, there are special appropriations from the central government for major public health programmes, including NIP, HIV/AIDS, tuberculosis (TB), malaria, etc. In 2024, the budgets allocated for NIP, HIV/AIDS and TB are US$456.04 million, US$980.74 million and US$179.34 million, respectively.^24^ Correspondingly, they covered 96.42 million children, 1.22 million people living with HIV^25^ and 0.50 million TB cases in 2022, respectively.^26^

**Figure 2:**
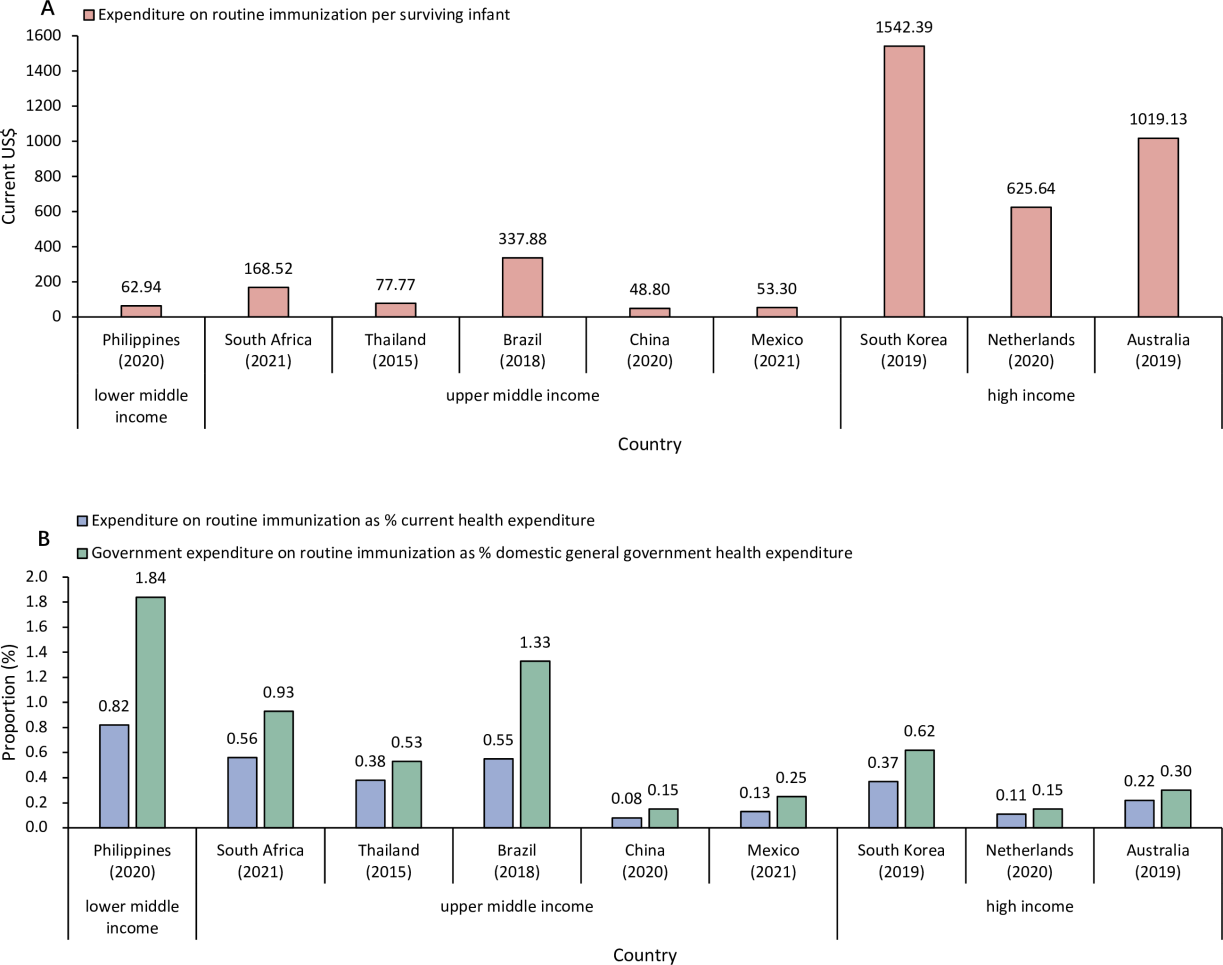
Expenditure on routine immunisation in China compared with selected Gavi-ineligible countries. In panel A, the denominator was the population aged 0 collected from the United Nations World Population Prospects 2022. In panel B, the denominators were collected from the World Bank. For expenditure in local currency, we used the exchange rate collected from the World Bank to convert it to US dollars. Within each income group, countries were listed in ascending order of GDP per capita in 2023 from left to right, based on World Bank data (current US$3725.6, US$6253.2, US$7171.8, US$10 043.6, US$12 614.1, US$13 926.1, US$33 121.4, US$62 536.7, US$64 711.8, respectively). Gavi, the Global Alliance for Vaccines and Immunisation; GDP, gross domestic product.

The funding for NIP in China is also low compared with the global average and many other MICs. The expenditure on routine immunisation per surviving infant in China was much lower than the global average of US$62 in 2017^27^ and the selected countries (box 1), even though half of them have a lower gross domestic product per capita (figure 2A). This result aligns with one study, which found that China’s estimated immunisation spending per surviving infant was far below the global average and many other upper-MICs such as Malaysia, Mexico, Russia, Thailand, South Africa and Brazil.^1^ Similarly, the share of current health expenditure allocated to routine immunisation and the share of domestic general government health expenditure dedicated to routine immunisation were both the lowest in China compared with other countries (figure 2B). Two major factors contributing to the differences in expenditure among countries are the varying vaccine prices and expensive vaccines introduced such as PCV and HPV.^27^ In our case, the price difference cannot completely explain the gap in immunisation financing between China and other countries. The prices of domestic vaccines included in China’s NIP are generally higher than those offered by the Pan American Health Organization (PAHO) and UNICEF, and many of the vaccine prices are slightly higher than the median price of self-procuring MICs (table 1). While regarding HPV and PCV vaccines, China has not introduced them, Thailand introduced the HPV vaccine and all the other selected countries introduced both vaccines in their NIPs.^28^

**Table 1:** Prices of vaccines included in the national immunisation programme in China compared with international organisations and other countries

Vaccine	PAHO (2024)	UNICEF (2024)	China (2024)	Median price in self-procuring countries (2022)	
				MICs	HICs
Bacille Calmette-Guérin (BCG)	US$0.18–0.83	US$0.14–0.25	US$0.69	US$0.2	US$2.2
Diphtheria-tetanus combined vaccine (DT)	US$0.18	US$0.18–0.21	US$0.09	US$0.2	US$0.6
Diphtheria-tetanus-acellular pertussis combined vaccine (DTaP)	US$19.29	–	US$0.81	US$17.4–26.1	US$19.2–31.6
Hepatitis A paediatric	US$8.12	US$7.55	US$3.11–4.53		
Hepatitis B paediatric	US$0.60	US$0.24–0.55	US$1.12	US$1.4	US$12.7
Inactivated polio vaccine (IPV)	US$1.50–5.50	US$1.25–3.37	US$4.94	US$3.1	US$12.2
Japanese encephalitis vaccine (JE)	–	US$0.45	US$1.38		
Measles-mumps-rubella combined vaccine (MMR)	US$1.71–5.30	US$1.71–3.56	US$3.50	US$4.5	US$8.5
Bivalent oral polio vaccine (bOPV)	US$0.13–0.18	–	US$0.37–0.38	US$0.2	US$0.3

Data shows the price per dose. Prices of vaccines with multiple products were shown in range. The PAHO prices were collected from PAHO Vaccine Prices 2024. The UNICEF prices were collected from UNICEF vaccine pricing data. Prices in China were collected from the Chinese Central Government Procurement website. The median prices in self-procuring countries were collected from the 2023 WHO Global Vaccine Market Report. The UNICEF price of hepatitis A paediatric vaccine was in 2021. The UNICEF price of JE vaccine was in 2023. We used the exchange rate collected from the World Bank to convert it to US dollars for prices in Chinese Yuan.

HICs, high-income countries; MICs, middle-income countries; PAHO, Pan American Health Organization.

### Restrictions about using health insurance funds for immunisation

Though widely used for NIP financing in many other countries, funding from social health insurance schemes in China is not allowed to pay for any public health programmes or services other than COVID-19 vaccines during the pandemic, including vaccines and immunisation services, as stipulated by the Healthcare Security Law of China.^17^ An example is that Chengdu City in Sichuan province allowed using medical insurance funds to pay for rabies vaccination before September 2021, but was then halted as it was deemed to be violating the law.^29^ According to the interview with a key informant from the national healthcare security authority, their current priority of health insurance fund use is still disease diagnosis and treatment instead of preventive health services.

With such a restriction, the only funding source for NIP is from the central and local governments through tax revenue. The expenditure on vaccine procurement is financed by the central government,^12 30^ and the administrative and operating expenses are jointly funded by the central and local governments. As the single source, government financing highly depends on local government funds. The central government provides 39% of the total funding and county-level governments account for 18% in 2015, only second to central government (no updated data available yet).^19^ This raises two concerns. First, the economic downturn after COVID-19 may exacerbate the financing stability of the governments. Second, there are enormous intercounty disparities due to the different fiscal capacities.^17^

Additionally, the proportion of vaccine procurement costs in routine immunisation in China is low. The percentage was 20.6% in 2015 and has increased since then.^19^ However, compared with the global average, it is still much lower as many countries spend around half of the total cost on vaccines and associated injection supplies.^19 31^ One important reason for this lower proportion of vaccine costs and the higher proportion of operational costs might be that China has not introduced comparatively more expensive PCV, HPV, Hib and Rotavirus vaccines yet.^31^

### High prices for non-NIP vaccines

The high price of non-NIP vaccines is another hurdle to expanding China’s NIP. According to the internal estimation of the Center for Disease Control and Prevention (CDC) of China, the technical and executive institution of NIP, the saved budget from the decreased number of newborns and lower prices due to pooled procurement of NIP vaccines is insufficient to introduce any new vaccines into NIP. Previous studies showed that prices of non-NIP vaccines in China are much higher than those offered by PAHO and UNICEF, and some are even higher than in the USA and Europe.^21 32^ Main reasons include the lack of negotiation power in procurement, unpredictable and unstable demand for vaccines and high marketing costs of manufacturers.^33^ The procurement of non-NIP vaccines is organised at the provincial level but operated at the county level, where negotiation power is limited. Manufacturers often collaborate to maintain prices across the region without reducing them in any province. As a result, the price of imported 13-valent PCV is around US$100 per dose.^34^ Even though the domestic products have lower prices of US$68.8–84.7,^34^ they are still much higher than the PAHO price of around US$13 and UNICEF MICs price of US$15–25 per dose.^35 36^

One consequence of the high price of non-NIP vaccines is the regional disparities in vaccine supply and coverage. Wealthier regions typically have sufficient fiscal budgets to introduce these new vaccines into their local immunisation programmes, offering free vaccinations to the target population. Even in the absence of such free programmes, more residents could afford to pay for the expensive vaccines out of pocket. While in underdeveloped areas with a higher burden of VPDs, the available non-NIP vaccines are limited, as indicated by some local CDCs, and the coverage rates are much lower due to limited government budgets and lower affordability of residents.^33^ In 2022, the provincial coverage rate of the first dose of HPV vaccine in females aged 9–45 in China ranged from 2.55% to 25.4%, with a difference of nearly 10 times.^37^

### Complicated and non-transparent decision-making mechanism to adjust NIP

The decision-making mechanism for new vaccine introduction is complicated and non-transparent, and the Ministry of Finance (MoF) was not involved in the initial discussion to plan for financing. Usually, the primary decision-makers for NIP expansion are the National Health Commission (NHC, formerly the Ministry of Health—MoH) and the National Disease Control and Prevention Administration (NDCPA), with the CDC’s technical support. The MoF is responsible for budget estimation and providing financial support after a policy is initially proposed. However, how the NHC and NDCPA make the decision, what evidence is used to support the decision-making and their roles and responsibilities in the decision-making process are unclear.

In comparison, countries with established National Immunisation Technical Advisory Groups (NITAGs) always rely on NITAGs to propose evidence-informed recommendations, and the MoH will make the final decision on immunisation policy change. While the socioeconomic development level in China has improved considerably in the past decades, the development progress of the governance system and capacity of NIP has lagged behind. China established its NITAG in 2017, though its functioning is still at an infancy stage and has not recommended the introduction of any vaccines yet.^17^ Key barriers may include a lack of adequate human and financial resources, low frequency of regular NITAG meetings, lack of long-term planning, the absence of clear guidelines for producing and using different types of evidence, etc.

Furthermore, the decision-making process is complicated by the influence of external factors and key stakeholders, including public opinion, key experts' influence, local practices, media advocacy, etc. However, the extent to which these factors impact decision-making and drive policy changes remains unclear and highly unknown to the public. For example, it is unclear how the high-level government made decisions on using health insurance funds to cover the costs of COVID-19 vaccines, the first time in history to use the fund for preventive health services.

## Opportunities for introducing new vaccines into NIP in China

There are opportunities to introduce new vaccines despite these challenges. Recent high-level documents in China emphasised the importance of preventing infectious diseases, highlighting the critical role of immunisation. In 2016, the State Council of China issued the Outline of Health China 2030 Planning, identifying NIP implementation and maintaining high vaccine coverage as one of the key strategic targets. Following this, the General Office of the State Council released a document on strengthening vaccine supplies and vaccination in 2017, advocating for the evidence-based introduction of new vaccines. According to NDCPA and NHC, China’s NIP supports catch-up vaccination for current vaccines up to age 18.^38^ Thus, it will be able to pay for adolescent vaccines, for example, HPV vaccine, without a change in law or regulation. Additionally, the experience gained from the COVID-19 vaccination campaign could be leveraged to facilitate the introduction of new vaccines, such as involving medical insurance funds and improving the immunisation information system.^39^

Although expanding NIP is a lengthy decision-making process, an increasing number of local governments have mobilised financial resources to add new vaccines to local immunisation programmes. For example, Weifang City in Shandong Province introduced a free dose of domestic 13-valent PCV in 2021 for children aged 6 months to 2 years.^40^ By August 2023, 10 provinces and 27 cities or counties implemented free or subsidised programmes, mostly school-based, to provide HPV vaccines for young girls.^41^ Some regions have also explored using health insurance funds to pay for non-NIP vaccines.^41^ These programmes have seen strong enthusiasm, leading to significant improvements in vaccination.^40 41^

The prices of some non-NIP vaccines have gradually decreased due to increased competition among domestic manufacturers and the improved procurement mechanism. For example, through competitive negotiation between two domestic companies and centralised procurement at the provincial level, the price of domestic 2-valent HPV vaccine has dropped by 92% from US$47.6 in 2019 to as low as US$3.9 per dose in Shandong Province in 2024.^41 42^ Combined with the significantly decreased fertility rate, this may present an opportune policy window for introducing new vaccines into NIP in the near future.

## Options for financing the NIP expansion in China

Considering the abovementioned opportunities, we propose the following options for sustainable financing for NIP expansion in China.

Increase funding for NIP expansion at both central and local governments. There are strong justifications considering the high return on investment of immunisation, the decreasing government expenditure on routine immunisation, the substantially lower investment in NIP compared with other MICs and regional disparities in vaccine access and coverage. One option is to adjust the government budget allocated to different public health programmes. With the successful control of some infectious diseases, the funding priority could shift away from diseases with relatively low burdens to NIP to optimise the efficiency of government investment. In addition, a considerable chunk of the public health funding allocated for two extensive public health programmes, HIV/AIDS and TB, has been used for diagnosis and treatment, which could be paid by the health insurance fund. In doing so, the released public health fund could be theoretically used for NIP expansion. Another option is to allocate more financial resources to underdeveloped areas or disadvantaged populations as a transitional strategy, which could help improve equity in immunisation services.

Diversify funding sources for immunisation financing, especially considering amending the Healthcare Security Law and allowing the use of health insurance funds to pay for or subsidise vaccinations. Robust evidence indicated that introducing many non-NIP vaccines is cost-effective or even cost-saving, reducing the avoidable diagnosis and treatment expenditure.^43^ Involving health insurance funds in NIP financing is a common practice in many other HICs and MICs (box 2). Drawing from these practices, China could add vaccinations to the benefits package and fully or partly reimburse them by the health insurance fund. The fact that China has used the health insurance fund to finance COVID-19 vaccines makes it promising for future policy change.^17^

Box 2 **Practices in other countries to finance routine immunisation through health insurance schemes**

High-income countries

- USA: Most private insurance plans cover recommended vaccines at no cost; military insurance covers all the recommended vaccines for people currently serving in the military; Medicare and Medicaid also cover many vaccines for children, and there may be a copay or fee.^45^
- Germany: Public health insurance is the primary source of vaccine financing, and all public health insurance companies pay for the vaccinations recommended by the Vaccination Commission.^46^
- Japan: Vaccination law requires subsidies for routine immunisation from national and local taxes, which are ultimately paid by Japan's national health insurance.^47^

Middle-income countries

- Indonesia: Immunisation is included in the benefit package of Indonesia’s national health insurance, with capitated payments to providers.^5^
- Thailand: Thailand purchases vaccines and immunisation services, including newborn vaccinations and vaccines covered by the National Expanded Programme on Immunisation, through its national health insurance scheme, the Universal Coverage Scheme.^48 49^

Support the domestic industry in developing and manufacturing non-NIP vaccines while improving the tendering strategy to optimise vaccine prices. Increasing the government budget for NIP is essential, but it is equally important to optimise the vaccine prices via feasible strategies to make the government investment more efficient. Good practices that proved effective in reducing prices for centralised procurement of drugs in China include combining the role of buyer and payer, increasing bargaining power by promising a large procurement volume across a broader market and establishing the procurement platform to improve data sharing and transparency, etc.^32^ That being said, a key lesson from the centralised procurement of drugs is to avoid lowering prices to a point where manufacturers are unwilling to produce, as this once led to stockouts of essential medicines.

Improve the decision-making mechanism for NIP expansion, streamlining the decision-making process and involving key financial stakeholders at the very beginning of discussions to develop feasible and sustainable financing strategies with high-quality data. A cross-ministerial coordination mechanism should be established with clear roles and responsibilities among key stakeholders, including NHC, NDCPA and MoF, with technical support from the NITAG. An evidence-informed approach should be used to collect and appraise key evidence to make decisions on new vaccine introduction. Meanwhile, clear guidance on the generation, presentation and utilisation of evidence should be established. The WHO has endorsed the Evidence-to-Recommendation framework developed by the US CDC and the Task Force for Global Health for the NITAG to propose evidence-informed recommendations, and one of the seven domains of the evidence used is resource use.^44^ Therefore, high-quality immunisation financing data need to be routinely collected and analysed to help develop financing strategies for future vaccine introduction.

## Conclusion

NIP financing in China is inadequate, which has hindered the introduction of new life-saving vaccines, especially compared with other domestic public health programmes and immunisation expenditures in other countries. With the high-level attention and experience gained from local pilot programmes, feasible options for actions should be seriously considered to optimise the financing model for NIP in China, including increasing government funding, diversifying financing channels such as using health insurance funds, supporting domestic vaccine manufacturers and improving procurement mechanisms and optimising the decision-making process for NIP expansion.

## Data Availability

Data are available in a public, open access repository.
