# Correction: Financing the introduction of new vaccines to the national immunisation programme in China: challenges and options for action

**DOI:** 10.1136/bmjgh-2024-017970corr1

**Published:** 2025-12-23

 Zhang X, Chen S, Zhu K, *et al.* Financing the introduction of new vaccines to the national immunisation programme in China: challenges and options for action. *BMJ Glob Health* 2025;10: e017970. doi:10.1136/bmjgh-2024–0 17 970

In the published version, it was identified that a few price data points in table 1 were incorrectly reported due to a miscalculation. In the China (2024) column, the price of BCG should be US$0.69 instead of US$3.44, and the price of DT should be US$0.09 instead of US$0.36. These errors are limited to this table and do not affect the study’s main findings, analyses, or conclusions.

**Table 1:** Prices of vaccines included in the national immunisation programme in China compared with international organisations and other countries

Vaccine	Paho (2024)	Unicef (2024)	China (2024)	Median price in self-procuring countries (2022)	
				MICs	HICs
Bacille Calmette-Guérin (BCG)	US$0.18–0.83	US$0.14–0.25	US$0.69	US$0.2	US$2.2
Diphtheria-tetanus combined vaccine (DT)	US$0.18	US$0.18–0.21	US$0.09	US$0.2	US$0.6
Diphtheria-tetanus-acellular pertussis combined vaccine (DTaP)	US$19.29	–	US$0.81	US$17.4–26.1	US$19.2–31.6
Hepatitis A paediatric	US$8.12	US$7.55	US$3.11–4.53		
Hepatitis B paediatric	US$0.60	US$0.24–0.55	US$1.12	US$1.4	US$12.7
Inactivated polio vaccine (IPV)	US$1.50–5.50	US$1.25–3.37	US$4.94	US$3.1	US$12.2
Japanese encephalitis vaccine (JE)	–	US$0.45	US$1.38		
Measles-mumps-rubella combined vaccine (MMR)	US$1.71–5.30	US$1.71–3.56	US$3.50	US$4.5	US$8.5
Bivalent oral polio vaccine (bOPV)	US$0.13–0.18	–	US$0.37–0.38	US$0.2	US$0.3

Data shows the price per dose. Prices of vaccines with multiple products were shown in range. The PAHO prices were collected from PAHO Vaccine Prices 2024. The UNICEF prices were collected from UNICEF vaccine pricing data. Prices in China were collected from the Chinese Central Government Procurement website. The median prices in self-procuring countries were collected from the 2023 WHO Global Vaccine Market Report. The UNICEF price of hepatitis A paediatric vaccine was in 2021. The UNICEF price of JE vaccine was in 2023. We used the exchange rate collected from the World Bank to convert it to US dollars for prices in Chinese Yuan.

HICs, high-income countries; MICs, middle-income countries; PAHO, Pan American Health Organisation.

